# Dexmedetomidine for Conscious Sedation and Controlled Hypotension in Head and Neck Surgery: A Single-Centre Experience

**DOI:** 10.3390/medicina62071232

**Published:** 2026-06-25

**Authors:** Ivana Vukušić, Borna Miličić, Ivan Šitum, Jerko Biloš, Igor Blivajs, Renata Curić Radivojević

**Affiliations:** 1 Department of Anaesthesiology, Resuscitation and Intensive Care, University Hospital Centre Zagreb, 10000 Zagreb, Croatia; ivana.vukusic@kbc-zagreb.hr (I.V.); ivan.situm@kbc-zagreb.hr (I.Š.); r.curicradivojevic@gmail.com (R.C.R.); 2 Department of Otolaryngology, Head and Neck Surgery, University Hospital Centre Zagreb, 10000 Zagreb, Croatia; jerko.bilos@kbc-zagreb.hr (J.B.); igor.blivajs@kbc-zagreb.hr (I.B.)

**Keywords:** dexmedetomidine, conscious sedation, local anaesthesia, head and neck surgery, controlled hypotension, monitored anaesthesia care, alpha-2 agonist, perioperative management

## Abstract

*Background and Objectives*: Elderly patients with head and neck tumours frequently present with multiple comorbidities and a potentially difficult airway, making general anaesthesia high-risk. Dexmedetomidine, a selective alpha-2 adrenoceptor agonist, provides conscious sedation without clinically significant respiratory depression, offering a compelling locoregional alternative. This study evaluated the haemodynamic profile, sedation kinetics, and satisfaction outcomes of a standardised dexmedetomidine-based protocol for head and neck surgery under local infiltration anaesthesia. *Materials and Methods*: A prospective, single-centre observational study was conducted at the University Hospital Centre Zagreb. Twenty-three consecutive adult patients received a continuous dexmedetomidine infusion at 0.5 μg/kg/h, initiated preoperatively in the post-anaesthesia care unit without a loading dose. Haemodynamic parameters, sedation-to-incision interval, cumulative dose, and postoperative patient and surgeon satisfaction (NRS 1–10) were recorded. Spearman rank-order correlation and the Mann–Whitney U test were used for statistical analysis. *Results*: The primary outcome of haemodynamic stability—defined as the absence of vasoactive or inotropic rescue—was achieved in all 23 patients (100%). The median cumulative dexmedetomidine dose was 52 μg (IQR 44–68 μg). Controlled hypotension was achieved in all patients, with a median nadir systolic blood pressure of 98 mmHg. Supplemental oxygen was required in only 2 of 23 patients (8.7%). Patient and surgeon satisfaction reached a median NRS score of 10 in both groups. The sedation-to-incision interval correlated with total drug dose (ρ = 0.74, *p* < 0.001), consistent with fixed-rate infusion pharmacokinetics. Hypertensive patients exhibited a greater reduction in systolic blood pressure (median 45 vs. 28 mmHg; *p* = 0.015). *Conclusions*: A fixed-rate dexmedetomidine infusion initiated in the post-anaesthesia care unit provides a feasible and potentially effective conscious sedation strategy for head and neck surgery under local infiltration anaesthesia in selected elderly and comorbid patients. In this pilot series, the protocol was associated with haemodynamic stability in all cases, low supplemental oxygen requirements, and high procedural satisfaction among both patients and surgeons. These findings are preliminary and require confirmation in larger, controlled studies.

## 1. Introduction

The incidence of head and neck tumours increases markedly with advancing age, a demographic trend that is well documented across European and global cancer registries [1,2,3]. This epidemiological reality means that a growing proportion of patients presenting for surgical resection of cutaneous malignancies, soft-tissue tumours, and other lesions of the head and neck region are elderly individuals with significant systemic comorbidity. The presence of multiple comorbidities—most commonly arterial hypertension, cardiac arrhythmias, diabetes mellitus, and impaired pulmonary reserve—substantially elevates the risk of adverse perioperative events when general anaesthesia is employed [4,5,6,7]. The additional challenge of a potentially difficult airway, encountered with particular frequency in this patient group, further augments anaesthetic risk and may itself serve as an independent indication for a locoregional approach [4,5].

For these reasons, locally potentiated anaesthesia (LPA) has been established for decades as the preferred technique for elective procedures on the head and neck in appropriately selected patients [8,9]. Surgical access is achieved through local infiltration of the operative field, while an intravenous sedative agent is concurrently administered to attenuate patient anxiety, ensure comfort, and improve intraoperative conditions. However, the sedative agents conventionally employed in this context—benzodiazepines, propofol, and opioids—each carry a clinically important liability: dose-dependent respiratory depression requiring supplemental oxygen delivery [10,11]. The administration of supplemental oxygen in proximity to active electrocautery introduces a well-recognised risk of operating-field ignition and facial burn injury [12,13,14,15]. This combination of circumstances has historically been one of the principal drivers of recourse to general anaesthesia even in patients for whom a locoregional technique would otherwise be preferable.

Dexmedetomidine is a highly selective agonist of alpha-2 adrenoceptors that has been in clinical use for several decades. Its pharmacological profile differs fundamentally from that of conventional sedative-hypnotics: rather than suppressing cortical activity through non-physiological mechanisms, dexmedetomidine induces sedation by activating endogenous sleep-promoting pathways centred on the locus coeruleus, producing a state analogous to non-REM sleep in which patients remain rousable and cooperative [16]. In clinically relevant doses, it does not cause significant respiratory depression, preserving spontaneous ventilation and airway reflexes [10,15]. It has also been reported to confer mild analgesic properties through spinal and supraspinal mechanisms, with some evidence of reduced opioid requirements in the perioperative period [17,18], although this effect was not a specific focus of the present study. The most clinically prominent adverse effects—bradycardia and arterial hypotension—are, to a moderate degree, therapeutically advantageous in elective head and neck surgery, where controlled reduction in blood pressure improves haemostasis and the clarity of the operative field [19,20].

Despite this favourable profile, the perioperative adoption of dexmedetomidine outside intensive care settings has been gradual, influenced by several converging factors. In the European Union, the European Medicines Agency marketing authorisation for dexmedetomidine (Dexdor^®^) is limited to ICU sedation, rendering perioperative use outside this context off-label in European centres [19]. In the United States, the FDA approved dexmedetomidine for procedural sedation of non-intubated patients prior to and during surgical and other procedures as early as 2008 [21], and its clinical applications have since expanded to procedural sedation, anaesthesia adjunct, and non-operating room settings [22,23]. Nevertheless, heterogeneity in dosing protocols, haemodynamic adverse effects, and the latency of the sedative effect at maintenance-only infusion rates without a loading dose have collectively limited wider adoption. Specifically, at rates of 0.2–0.5 μg/kg/h without a loading dose, adequate sedation depth is typically not achieved for 20 to 40 min [19,21]—a deliberate protocol-specific trade-off between haemodynamic safety and onset speed in elderly comorbid patients, rather than an inherent pharmacological limitation of the agent itself. The operating theatre represents a bottleneck in surgical workflow, and any extension of induction or recovery time directly reduces throughput and programme efficiency. To circumvent this logistical obstacle, we implemented a preoperative initiation protocol in which the dexmedetomidine infusion is commenced in the post-anaesthesia care unit (PACU) prior to transfer to the operating theatre. This approach allows the sedative equilibration period to be absorbed within the preparation phase, without consuming scheduled operating time. Body weight was the sole determinant of infusion rate under the fixed-dose protocol (0.5 μg/kg/h), facilitating straightforward and reproducible administration by nursing staff. The present study reports the clinical outcomes of this protocol in a consecutive series of patients with head and neck lesions.

## 2. Materials and Methods

### 2.1. Study Design and Patient Population

This prospective, single-centre observational study was conducted at the University Hospital Centre Zagreb. A consecutive series of 23 adult patients undergoing elective cutaneous and soft-tissue excision and reconstruction of the head and neck region under local infiltration anaesthesia with dexmedetomidine-based conscious sedation were enrolled. Inclusion criteria were: adult patients (≥18 years) scheduled for elective cutaneous or soft-tissue excision and reconstruction of the head and neck region; joint determination by the attending surgeon and anaesthesiologist that a locoregional technique was preferable to general anaesthesia; and absence of contraindications to dexmedetomidine (known hypersensitivity, pre-existing advanced atrioventricular block not supported by a pacemaker, or uncontrolled haemodynamic instability). The clinical indication for locoregional anaesthesia was based on one or more of the following: advanced patient age, systemic comorbidity (ASA ≥ II), or potentially difficult airway anatomy. Exclusion criteria included: patient refusal of locoregional anaesthesia, inability to provide informed consent, and emergency or oncologically urgent procedures. The study was conducted in accordance with the principles of the Declaration of Helsinki, and patient consent was obtained as part of routine preoperative assessment.

### 2.2. Anaesthetic Protocol

All patients received dexmedetomidine (Dexdor^®^, Orion Pharma, Espoo, Finland)) as a continuous intravenous infusion at a fixed rate of 0.5 μg/kg/h, prepared at a concentration of 4 μg/mL in 0.9% sodium chloride. The infusion rate of 0.5 μg/kg/h was selected on the basis of three considerations: (i) it represents the lower end of the published maintenance dose range for dexmedetomidine-based monitored anaesthesia care (0.2–1.0 μg/kg/h) [21], providing adequate depth of sedation with a favourable haemodynamic safety margin in a predominantly elderly and hypertensive population—notably, the FDA prescribing information explicitly recommends dose reduction in patients aged over 65 years, in whom hypotension occurs at substantially higher rates [21]; (ii) randomised controlled trials in comparable head and neck surgical settings (functional endoscopic sinus surgery) have employed maintenance doses spanning 0.2–0.8 μg/kg/h [24,25,26,27,28], and 0.5 μg/kg/h represents a deliberate middle point within this range, balancing adequate sedation with haemodynamic safety in a comorbid elderly cohort; and (iii) at this rate, omission of a loading dose results in a predictable equilibration period of approximately 20–40 min [19], which is absorbed within the PACU preparation phase without consuming scheduled operative time. No loading dose was administered. The infusion was initiated in the PACU prior to transfer to the operating theatre, allowing a pre-incision equilibration period of variable duration. The infusion was temporarily suspended whenever a clinically significant haemodynamic response was observed, defined as a systolic arterial pressure below 90 mmHg or a reduction exceeding 30% from preoperative baseline—thresholds consistent with current perioperative cardiovascular guidelines [17,18] and established definitions of controlled hypotension in head and neck surgery [29,30]; the infusion was recommenced once haemodynamic recovery was confirmed. Supplemental oxygen was administered only when clinically indicated; its routine omission in procedures involving electrosurgery was intentional, in order to minimise the risk of operating-field ignition.

Multimodal analgesia was administered according to a standardised adjunct protocol. Metamizole 2.5 g and dexamethasone 8 mg were administered intravenously in selected cases; sufentanil 5–10 μg, paracetamol 1 g, or an NSAID (ketoprofen 100 mg or ibuprofen 400 mg) were added at the anaesthesiologist’s discretion. Surgical-site infiltration with lidocaine 2%, with or without adrenaline (1:80,000–1:200,000), was performed by the operating surgeon immediately prior to incision; lidocaine with adrenaline was used in 18 of 23 cases (78.3%).

### 2.3. Monitoring and Outcome Measures

Standard intraoperative monitoring was applied throughout each procedure, comprising continuous non-invasive arterial blood pressure measurements at five-minute intervals, continuous pulse oximetry, and three-lead electrocardiography. The primary outcome was haemodynamic stability, operationally defined as the absence of any requirement for vasoactive or inotropic pharmacological rescue throughout the operative period. The haemodynamic thresholds that would trigger infusion suspension and, if necessary, vasoactive intervention were pre-specified in the anaesthetic protocol (Section 2.2): a systolic arterial pressure below 90 mmHg or a reduction exceeding 30% from the preoperative baseline. Additional haemodynamic variables—including nadir systolic blood pressure, absolute blood pressure reduction, and nadir heart rate—were recorded as descriptive monitoring parameters to characterise the haemodynamic trajectory of the protocol; these are distinct from the pre-specified primary and secondary outcomes. Secondary outcomes comprised: (i) the sedation-to-incision interval; (ii) total cumulative dexmedetomidine dose; (iii) patient-reported procedural satisfaction on a Numerical Rating Scale (NRS, 1–10); and (iv) surgeon-reported intraoperative conditions on an identical NRS instrument.

### 2.4. Statistical Analysis

Given the small sample size (N = 23) and non-Gaussian distributional characteristics of the primary outcome variables, exclusively nonparametric statistical methods were employed. Continuous variables are reported as median with interquartile range (IQR) and full range; categorical variables as absolute frequencies and proportions. Monotonic associations were evaluated using Spearman’s rank-order correlation coefficient (ρ). Between-group comparisons were performed using the two-sided Mann–Whitney U test and the Kruskal–Wallis H test. Statistical significance was defined as *p* < 0.05 (two-tailed). All analyses were performed using IBM SPSS Statistics, Version 29.0 (IBM Corp., Armonk, NY, USA). Given the pilot and exploratory design of the study, no a priori sample size calculation was performed and no correction for multiple comparisons was pre-specified. The sample size of 23 patients is consistent with published guidance on pilot study methodology, which supports the use of smaller samples when the primary aim is to assess feasibility and generate preliminary data rather than to test hypotheses definitively [31,32,33]. The comparison of systolic blood pressure reduction between hypertensive and normotensive patients was a post hoc, unplanned exploratory analysis. All results should therefore be interpreted as hypothesis-generating rather than confirmatory.

## 3. Results

### 3.1. Patient Characteristics

The results are reported in the following order: patient characteristics (Section 3.1), pharmacokinetic parameters (Section 3.2 and Section 3.3), the primary outcome—haemodynamic stability (Section 3.4)—followed by subgroup analyses and satisfaction data (Section 3.5, Section 3.6 and Section 3.7). Section 3.2 and Section 3.3 describe descriptive pharmacokinetic variables and are presented first for narrative coherence; the primary efficacy endpoint is formally reported in Section 3.4. The majority were classified as ASA Physical Status III (*n* = 16, 69.6%), with six patients (26.1%) classified as ASA II and one (4.3%) as ASA III–IV. Arterial hypertension was the predominant comorbidity, present in 19 patients (82.6%). Diabetes mellitus was documented in 7 patients (30.4%), atrial fibrillation in 5 (21.7%), active smoking in 4 (17.4%), and regular alcohol use in 4 (17.4%). Long-term polypharmacy was documented in 21 patients (91.3%). Full baseline characteristics are presented in Table 1.

The study cohort comprised 23 patients (10 male, 13 female), with a median age of 76 years (IQR 67–80; range 50–85). The majority were classified as ASA Physical Status III (n = 16, 69.6%), with six patients (26.1%) classified as ASA II and one (4.3%) as ASA III–IV. Arterial hypertension was the predominant comorbidity, present in 19 patients (82.6%). Diabetes mellitus was documented in 7 patients (30.4%), atrial fibrillation in 5 (21.7%), active smoking in 4 (17.4%), and regular alcohol use in 4 (17.4%). Long-term polypharmacy was documented in 21 patients (91.3%). Full baseline characteristics are presented in Table 1.

### 3.2. Dexmedetomidine Dosing and Sedation-to-Incision Interval

**Figure 1:**
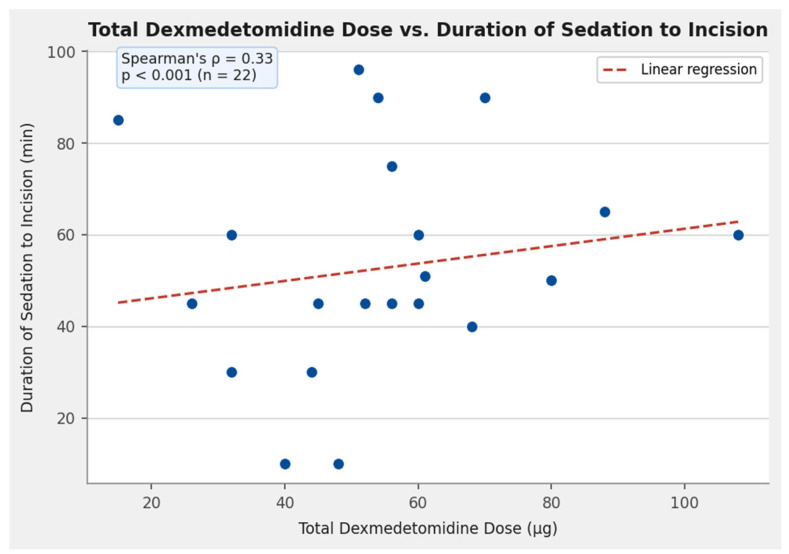
Scatterplot of total dexmedetomidine dose (μg) against sedation-to-incision interval (min). Spearman’s ρ = 0.74, *p* < 0.001; *n* = 22. Each point represents an individual patient

**Figure 2:**
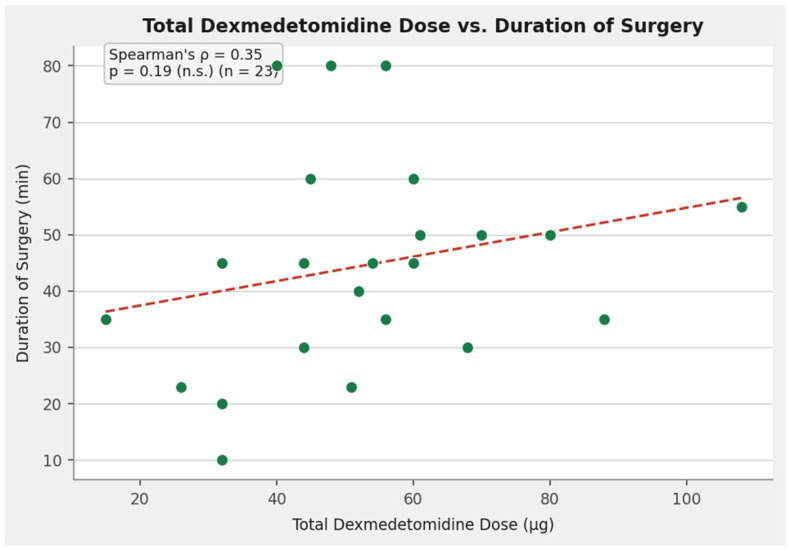
Scatterplot of total dexmedetomidine dose (μg) against duration of surgery (min). Spearman’s ρ = 0.29, *p* = 0.19; *n* = 23. Each point represents an individual patient.

The total cumulative dose of dexmedetomidine ranged from 15 to 108 μg (median 52 μg, IQR 44–68 μg). The sedation-to-incision interval had a mean of 50.5 min (median 50 min, IQR 40–75; range 10–96 min). A strong, statistically significant positive correlation was identified between cumulative drug dose and duration of the pre-incision sedation interval (Spearman’s ρ = 0.74, *p* < 0.001; Figure 1), consistent with the continuous infusion pharmacokinetics of dexmedetomidine administered without a loading dose. No significant association was observed between total dose and surgical duration (ρ = 0.29, *p* = 0.19; Figure 2).

### 3.3. Association Between Dose and Body Weight

**Figure 3:**
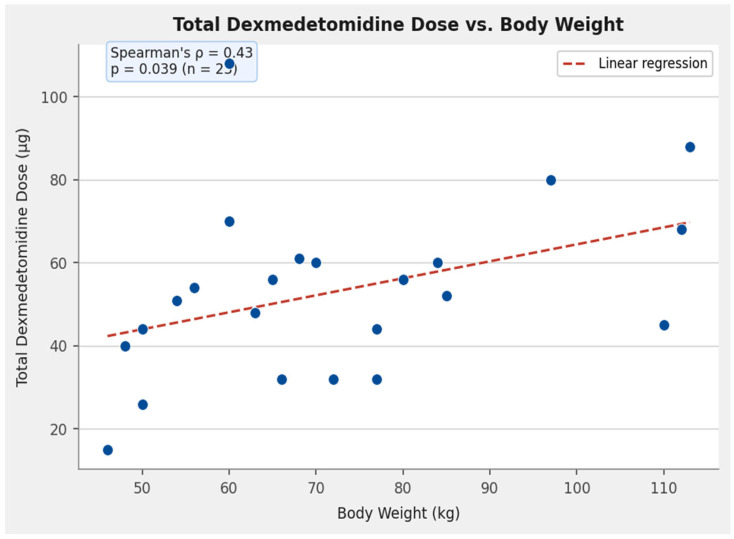
Scatterplot of total dexmedetomidine dose (μg) against patient body weight (kg). Spearman’s ρ = 0.43, *p* = 0.039; *n* = 23. Each point represents an individual patient.

A statistically significant positive correlation was identified between the total administered dexmedetomidine dose and patient body weight (Spearman’s ρ = 0.43, *p* = 0.039; Figure 3). This finding is clinically coherent, given that the infusion rate was prescribed at a fixed weight-based dose of 0.5 μg/kg/h. No significant correlation was identified between the total dose and BMI (ρ = 0.37, *p* = 0.083), indicating that the dose-weight relationship is driven by lean body mass rather than adiposity-adjusted body habitus. No significant correlations were identified for age (ρ = −0.17, *p* = 0.44), baseline systolic blood pressure (ρ = 0.13, *p* = 0.57), or baseline heart rate (ρ = 0.13, *p* = 0.57).

### 3.4. Haemodynamic Profile

The primary outcome of haemodynamic stability—defined as the absence of any requirement for vasoactive or inotropic pharmacological rescue—was achieved in all 23 patients (100%). No patient required pharmacological intervention for haemodynamic instability at any point during the procedure. This finding constitutes the principal efficacy endpoint of the study. Controlled hypotension was achieved in all patients. The median nadir systolic arterial pressure was 98 mmHg (IQR 90–120; range 72–135 mmHg), representing a median absolute reduction of 38 mmHg (IQR 25–55; range 8–100 mmHg) from preoperative baseline. The median baseline heart rate was 65 beats/min (IQR 57–77), with a median nadir of 52 beats/min (IQR 45–60) and a median decrease of 12 beats/min (IQR 8–18; range 3–29 beats/min).

**Figure 4:**
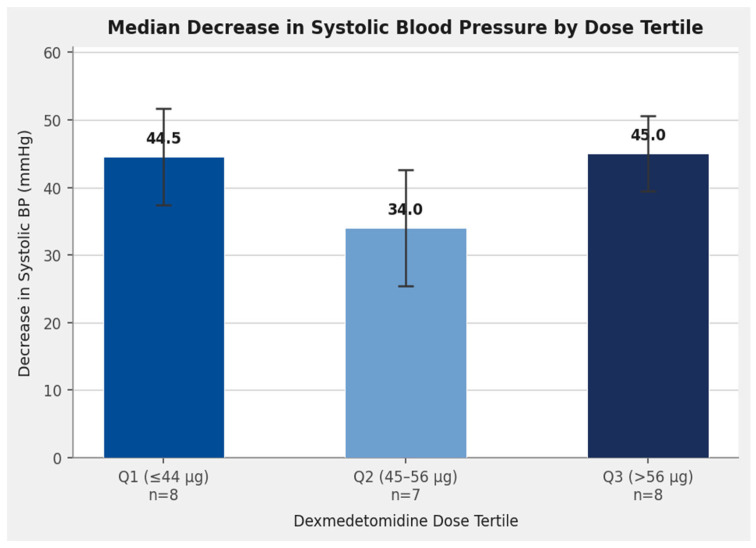
Median decrease in systolic arterial blood pressure (mmHg) stratified by tertiles of total dexmedetomidine dose. Spearman’s ρ = 0.002, *p* = 0.99.

**Figure 5:**
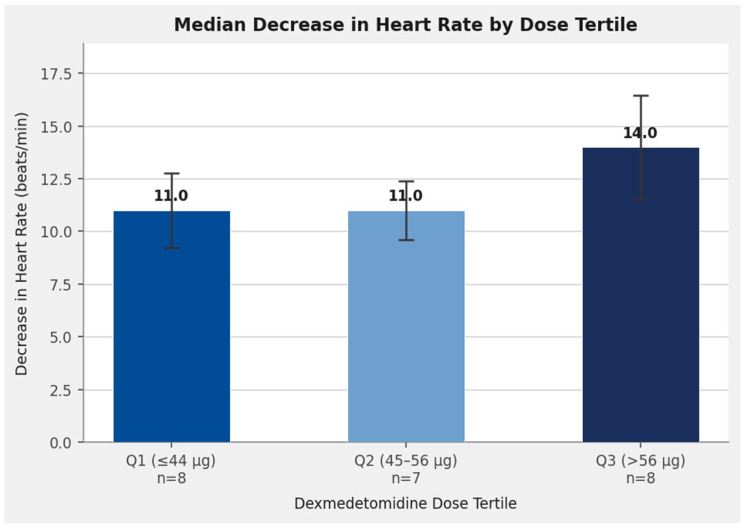
Median decrease in heart rate (beats/min) stratified by tertiles of total dexmedetomidine dose. Spearman’s ρ = 0.31, *p* = 0.15.

**Figure 6:**
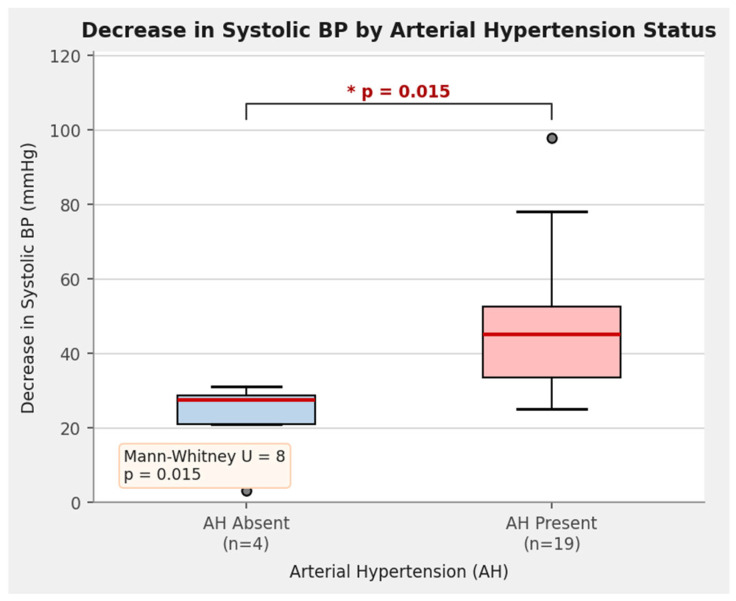
Distribution of decrease in systolic blood pressure (mmHg) stratified by arterial hypertension status. Hypertensive vs. normotensive patients: median 45 vs. 28 mmHg; Mann–Whitney U, *p* = 0.015. * denotes statistical significance (*p* < 0.05, Mann–Whitney U test). The red horizontal line represents the median; boxes indicate the interquartile range, whiskers represent values within 1.5 × IQR, and black dots indicate outliers. The asterisk denotes a statistically significant difference between groups (*p* = 0.015, Mann–Whitney U test).

Stratification of the blood pressure and heart rate reductions by tertiles of total administered dose was performed as an exploratory visualisation approach; tertile-based grouping was chosen in preference to continuous regression analysis to allow graphical representation of dose–response trends in a small sample (*n* = 23), where continuous regression may be disproportionately influenced by individual outliers. This method does not imply a hypothesis of threshold-dependent effects. This approach did not reveal statistically significant dose-dependent gradients (systolic BP decrease: ρ = 0.002, *p* = 0.99; heart rate decrease: ρ = 0.31, *p* = 0.15). However, hypertensive patients exhibited a substantially greater median decrease in systolic blood pressure (45 mmHg; IQR 28–58) than normotensive patients (28 mmHg; IQR 13–35), a difference that attained statistical significance (Mann–Whitney U, *p* = 0.015; Figure 4, Figure 5 and Figure 6). No patient required vasoactive or inotropic pharmacological intervention. Supplemental oxygen was required in only 2 patients (8.7%).

### 3.5. Subgroup Analyses

**Figure 7:**
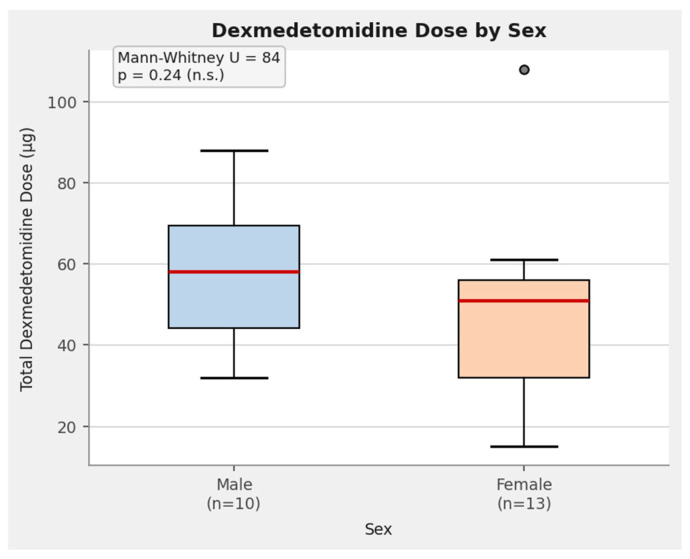
Distribution of total dexmedetomidine dose (μg) stratified by sex. Mann–Whitney U, *p* = 0.24.

**Figure 8:**
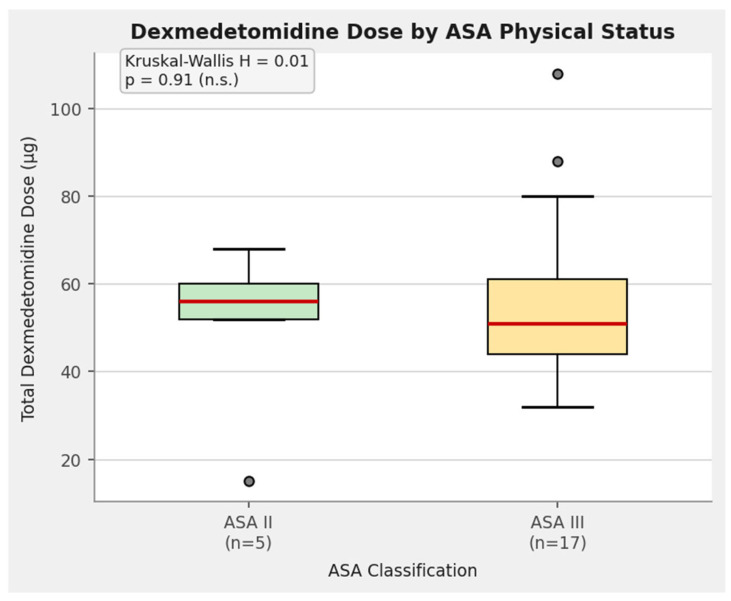
Distribution of total dexmedetomidine dose (μg) stratified by ASA physical status. Kruskal–Wallis H = 0.77, *p* = 0.38.

Dose distribution was comparable between male and female patients (median 58 vs. 51 μg; Mann–Whitney U, *p* = 0.24) and between ASA II and ASA III patients (Kruskal–Wallis H = 0.77, *p* = 0.38). The presence of arterial hypertension, atrial fibrillation, diabetes mellitus, smoking, or alcohol use did not significantly influence the total dexmedetomidine dose administered (all *p* > 0.60) (Figure 7 and Figure 8).

### 3.6. Patient and Surgeon Satisfaction

**Figure 9:**
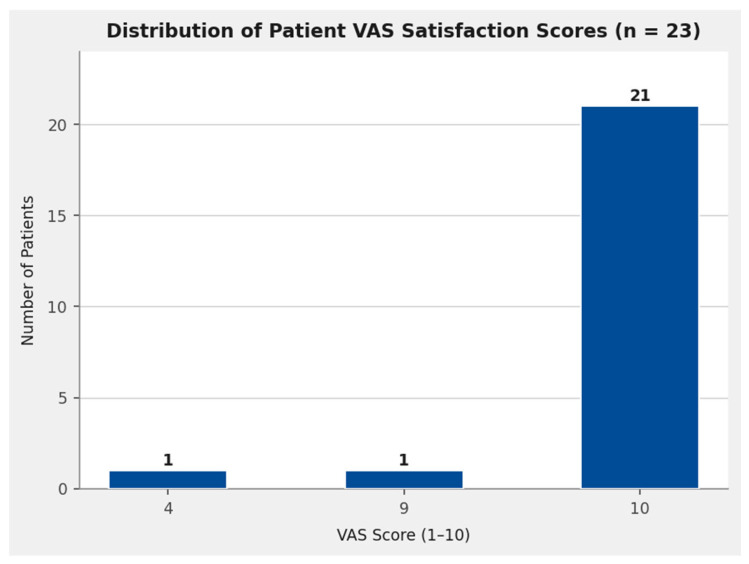
Frequency distribution of patient-reported NRS satisfaction scores (1–10). *n* = 23.

**Figure 10:**
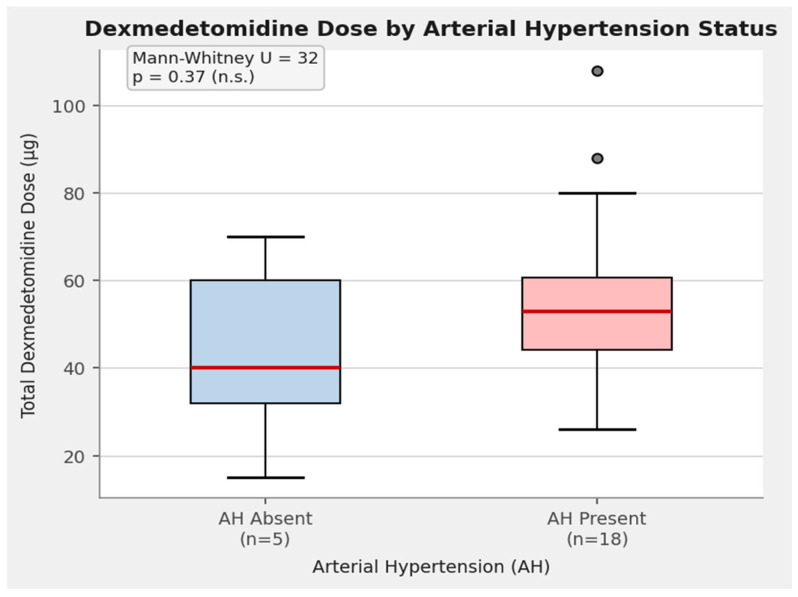
Distribution of total dexmedetomidine dose (μg) by arterial hypertension status. *p* = 0.63.

Patient-reported NRS satisfaction demonstrated a pronounced ceiling effect: 21 of 23 patients (91.3%) assigned the maximum score of 10. The cohort median was 10 (IQR 10–10; range 4–10). Surgeon-reported intraoperative conditions were equivalent, with a median of 10 (IQR 10–10; range 6–10). The overall mean satisfaction score, aggregated across both patient and surgeon ratings, was 9.7 out of 10. No statistically significant associations with total dexmedetomidine dose were identified for either instrument (patient NRS: ρ = −0.27, *p* = 0.22; surgeon NRS: ρ = 0.08, *p* = 0.73) (Figure 9 and Figure 10).

### 3.7. Summary of Haemodynamic and Satisfaction Parameters

A consolidated summary of haemodynamic monitoring parameters, dosing variables, and satisfaction outcomes is presented in Table 2 for ease of reference. These variables include both the pre-specified primary outcome (haemodynamic stability, achieved in 100% of patients) and the descriptive secondary and exploratory parameters reported in Section 3.2, Section 3.3, Section 3.4, Section 3.5 and Section 3.6.

## 4. Discussion

This prospective single-centre study demonstrates that a fixed-rate dexmedetomidine infusion at 0.5 μg/kg/h, initiated in the post-anaesthesia care unit and combined with surgical-site infiltration of lidocaine, provides effective conscious sedation and haemodynamically favourable operative conditions for elective head and neck procedures in a comorbid elderly population. The absence of any requirement for vasoactive or inotropic rescue in all 23 patients, in conjunction with a median nadir systolic blood pressure of 98 mmHg, confirms that the protocol achieves controlled hypotension within a clinically safe range [1,2].

The strong positive correlation between total dexmedetomidine dose and the sedation-to-incision interval (ρ = 0.74, *p* < 0.001) reflects the mathematical coupling inherent to a fixed-rate continuous infusion administered without a loading dose: cumulative dose is, by definition, the product of infusion rate and duration, and a longer pre-incision equilibration period necessarily results in a higher total dose administered. This correlation therefore validates the internal consistency and reproducibility of the dosing protocol across all 23 patients—confirming that the infusion was administered as prescribed—but should not be interpreted as a measure of clinical efficacy or sedation depth. The clinically meaningful indicators of protocol utility are the haemodynamic outcomes, oxygen supplementation rate, and patient and surgeon satisfaction scores reported in Section 3.4, Section 3.5 and Section 3.6. By commencing the infusion in the PACU, the equilibration period is removed from the operating theatre schedule entirely, which remains the principal logistical advantage of the preoperative initiation strategy [19,21]. The significant correlation between total dose and body weight (ρ = 0.43, *p* = 0.039) and the contrasting absence of a significant correlation with BMI (ρ = 0.37, *p* = 0.083) reflect the weight-based rather than adiposity-adjusted nature of the dosing protocol. It should be acknowledged, however, that the difference in statistical significance between these two correlations may partly reflect the limited statistical power of the present study. With a sample of 23 patients, the study is insufficiently powered to reliably distinguish between a true dose-body weight relationship and a dose-BMI relationship of comparable effect size; the observed divergence in *p*-values may represent a Type II error rather than a genuine pharmacokinetic difference. In obese patients in particular, where lean body mass constitutes a smaller proportion of total body weight, weight-based dosing at 0.5 μg/kg/h may result in relative overdosing with respect to pharmacologically active tissue mass. Several authors have proposed that lean body mass or fat-free mass is a superior dosing scalar for dexmedetomidine compared with total body weight: pharmacokinetic studies demonstrate that the drug’s clearance and volumes of distribution are best described by lean body weight-based models, with adipose tissue contributing negligibly to clearance [19,34,35,36]. Future studies with larger cohorts and prospective anthropometric phenotyping should specifically evaluate whether lean body mass-adjusted dosing improves the predictability of the sedation-to-incision interval and reduces haemodynamic variability in obese patients.

Notably, patients with pre-existing arterial hypertension exhibited a significantly greater reduction in systolic blood pressure (median 45 mmHg) than normotensive patients (median 28 mmHg; *p* = 0.015). This augmented response is pathophysiologically consistent with the mechanism of action of alpha-2 agonists: hypertensive patients characteristically exhibit elevated baseline sympathetic nervous system activity, and selective inhibition of central sympathetic outflow through locus coeruleus alpha-2 receptors produces a proportionally greater reduction in peripheral vascular resistance and cardiac output in this group [2,9,10].

The low requirement for supplemental oxygen—observed in only 2 of 23 patients (8.7%)—represents a clinically significant and, in the authors’ view, underappreciated safety advantage of dexmedetomidine over conventional sedative regimens in the specific context of head and neck electrosurgery. Operating room fires, though relatively uncommon in absolute terms, are a disproportionately catastrophic adverse event: the head and neck surgical field constitutes a recognised high-risk environment because oxygen-enriched atmosphere, an ignition source (monopolar electrocautery), and combustible material (draping, hair, and mucosal surfaces) coexist in immediate proximity [12,13,14,15]. The American Society of Anesthesiologists Task Force on Operating Room Fires has explicitly designated the face and neck as a high-risk anatomical site for surgical fire, with supplemental oxygen delivery identified as the most readily modifiable risk factor [12,13]. Benzodiazepines, propofol, and opioids routinely require oxygen supplementation to maintain acceptable arterial saturation, creating a continuous fire hazard throughout the procedure [10,11,12,13,14,15]. By contrast, dexmedetomidine’s preservation of spontaneous ventilation and airway reflexes at clinically relevant doses permits the routine omission of supplemental oxygen in the overwhelming majority of patients, eliminating the oxygen-enrichment component of the fire triangle. In a series of 23 patients undergoing electrosurgical procedures of the head and neck, only 2 (8.7%) required supplemental oxygen—in both cases for brief periods attributable to transient desaturation unrelated to respiratory depression. This rate compares favourably with published case series of propofol- or midazolam-based sedation, in which oxygen supplementation is universal [10,11]. We propose that the ability to omit supplemental oxygen in the majority of cases should be considered a clinically meaningful advantage of dexmedetomidine in head and neck electrosurgical practice, and that this consideration merits prospective evaluation in comparative studies. It should be noted, however, that the present study did not employ capnographic monitoring, and respiratory rate was not systematically recorded as a prospective study variable beyond the binary outcome of supplemental oxygen requirement. We wish to address this directly. The ASA Practice Guidelines for Moderate Procedural Sedation and Analgesia (2018) recommend capnography “unless precluded or invalidated by the nature of the patient, procedure, or equipment” [37]—language also used in the ASA Standards for Basic Anaesthetic Monitoring. Head and neck surgery under local infiltration anaesthesia in an open surgical field involves frequent head repositioning, surgical manipulation adjacent to the airway, and the presence of blood and secretions, all of which compromise the reliability of sidestream nasal capnography in this setting. Recent evidence confirms that nasal cannula capnography fails to capture a substantial proportion of respiratory cycles during oral breathing in open-airway surgical patients, with false-positive rates for apnoea as high as 88% [38,39]. Our monitoring protocol—continuous pulse oximetry with alarms, continuous clinical observation of respiratory pattern and chest excursion by a dedicated anaesthesiologist, and maintained verbal contact with all patients (consistent with Ramsay Sedation Scale 2–3)—satisfies the ASA standard of “continually monitoring ventilatory function by observation of qualitative clinical signs” in conjunction with continuous pulse oximetry. The added value of capnography in low-risk patients under moderate sedation is itself debated: a 2019 systematic review concluded that “recent high-quality studies… found no benefit with addition of capnography” in this context [40], a view echoed by the ASGE sedation guidelines [41]. Furthermore, the pharmacological profile of dexmedetomidine at the doses employed provides mechanistic reassurance: the FDA prescribing information confirms that at 0.2–0.7 μg/kg/h “respiratory rate and oxygen saturation remained within normal limits and there was no evidence of respiratory depression” in volunteer studies [21]; crossover pharmacodynamic studies demonstrate that, unlike opioids, dexmedetomidine does not cause clinically significant respiratory depression [40]; and a randomised trial in oral and maxillofacial surgery documented respiratory events requiring intervention in only 2.7% of dexmedetomidine-treated patients versus 25.4% in the propofol–fentanyl group [41]. Clinically, SpO_2_ remained above 94% in all patients throughout all procedures as confirmed by continuous pulse oximetry, and no patient required airway intervention, bag-mask ventilation, oropharyngeal airway insertion, or conversion to general anaesthesia. We acknowledge, however, that dexmedetomidine does attenuate ventilatory responses to hypoxia and hypercapnia to a degree comparable to propofol at equivalent sedation depths [42,43,44], and that respiratory safety should not be overstated. Future studies should consider incorporating combined nasal–oral capnography sampling systems, where technically feasible, to provide more granular objective respiratory data [38].

Atrial fibrillation, present in five patients (21.7%), did not result in any clinically significant arrhythmic event or the requirement for pharmacological rate control beyond the baseline regimen. This observation is consistent with the approved prescribing information, which indicates that dexmedetomidine is safe in patients with haemodynamically compensated atrial fibrillation in the absence of pre-existing advanced AV block [2,7].

Patient-reported NRS satisfaction demonstrated a pronounced ceiling effect, with 91.3% of patients awarding the maximum score of 10. Surgeon satisfaction was equally high. Dexmedetomidine addresses the principal determinants of surgical satisfaction in locoregional practice: its anxiolytic and sedative properties produce a calm, responsive patient; its sympatholytic effect reduces capillary ooze and improves tissue planes; and the low rate of haemodynamic complications minimises the frequency of surgical pauses [3,4,5,6].

This study has several limitations that warrant consideration. First, the sample size of 23 patients, while sufficient for exploratory analyses, limits statistical power and precludes definitive conclusions regarding subgroup effects, dosing optimisation, and predictors of haemodynamic response. Second, the single-centre design restricts the generalisability of the findings to institutions with comparable patient populations and anaesthetic infrastructure. Third, and most fundamentally, the study lacks a concurrent control group. The absence of randomisation means that the observed outcomes cannot be causally attributed to the dexmedetomidine protocol in isolation, and comparisons with conventional sedative regimens must rely on historical or published reference data. A randomised controlled trial comparing dexmedetomidine-based conscious sedation with a standard midazolam–fentanyl or propofol-based regimen would represent the methodologically optimal approach to definitively quantifying the haemodynamic, respiratory, and safety advantages suggested by the present data. We acknowledge that such a trial was not feasible in the present context: the study was conducted as a prospective observational evaluation of an institutional protocol already in clinical use, and the attending anaesthesiologist’s clinical judgement that dexmedetomidine was the preferred agent for each enrolled patient precluded equipoise-based randomisation. Historical control data from comparable head and neck surgery populations consistently report oxygen supplementation rates approaching 100% with conventional sedative agents [10,11], providing contextual reference for the 8.7% rate observed in the present series. Comparative randomised data further contextualise the present findings: a Phase III RCT of dexmedetomidine for monitored anaesthesia care (n = 326) demonstrated significantly lower rescue sedative requirements and higher patient satisfaction compared with placebo [45]. These external data provide hypothesis-generating comparative context pending formal controlled evaluation of the present protocol. Larger, randomised, multicentre trials are warranted to confirm and extend these findings. Fourth, no a priori sample size calculation was performed; the study is therefore underpowered to detect differences in secondary endpoints with conventional statistical reliability, and all subgroup analyses—including the comparison of systolic blood pressure reduction between hypertensive and normotensive patients (*p* = 0.015)—should be regarded as exploratory and hypothesis-generating, pending replication in an adequately powered prospective cohort. Fifth, 18 of 23 patients (78.3%) received local anaesthetic with epinephrine (1:80,000–1:200,000), which may have contributed to haemostasis and influenced haemodynamic parameters; the observed outcomes therefore reflect the combined effect of the multimodal protocol rather than dexmedetomidine in isolation. Future studies should stratify prospectively by local anaesthetic preparation, or include a vasoconstrictor-free comparator arm, to isolate the haemodynamic contribution of dexmedetomidine. Finally, the study was not prospectively registered in a publicly accessible clinical trials registry. As the study is observational and non-interventional, prospective registration is not mandated under ICMJE guidelines; however, future studies of this protocol should adopt prospective registration to enhance transparency and reproducibility.

## 5. Conclusions

A fixed-rate dexmedetomidine infusion at 0.5 μg/kg/h, initiated in the post-anaesthesia care unit prior to transfer to the operating theatre, represents a logistically practical and clinically promising sedation strategy for elective head and neck surgery under local infiltration anaesthesia in elderly, comorbid patients. In this single-centre pilot series, the protocol was associated with haemodynamic stability in all cases, low supplemental oxygen requirements, and uniformly high patient and surgeon satisfaction. The pharmacodynamic response appeared amplified in patients with arterial hypertension, a finding consistent with the mechanism of action of alpha-2 agonists in this population. These observations are hypothesis-generating; definitive conclusions regarding superiority over alternative sedation approaches require confirmation in larger, randomised, controlled trials. The preoperative PACU-initiation strategy merits prospective evaluation as a means of overcoming the principal logistical barrier to wider perioperative adoption of dexmedetomidine in head and neck surgical practice.

## Figures and Tables

**Table 1:** Baseline demographic, anthropometric, and clinical characteristics of the study cohort.

Characteristic	Total (*N* = 23)	Male (*n* = 10)	Female (*n* = 13)
Age, years, median (IQR)	76 (67–80)	73 (63–80)	77 (70–80)
Body weight, kg, median (IQR)	66 (56–85)	82 (68–112)	63 (50–77)
BMI, kg/m^2^, median (IQR)	24.6 (22.5–30.0)	26.1 (23.4–31.8)	23.7 (22.2–29.4)
ASA II, *n* (%)	6 (26.1%)	3 (30.0%)	3 (23.1%)
ASA III, *n* (%)	16 (69.6%)	7 (70.0%)	9 (69.2%)
ASA III–IV, *n* (%)	1 (4.3%)	0 (0%)	1 (7.7%)
Arterial hypertension, *n* (%)	19 (82.6%)	8 (80.0%)	11 (84.6%)
Atrial fibrillation, *n* (%)	5 (21.7%)	2 (20.0%)	3 (23.1%)
Diabetes mellitus, *n* (%)	7 (30.4%)	3 (30.0%)	4 (30.8%)
Active smoker, *n* (%)	4 (17.4%)	2 (20.0%)	2 (15.4%)
Regular alcohol use, *n* (%)	4 (17.4%)	3 (30.0%)	1 (7.7%)
Long-term polypharmacy, *n* (%)	21 (91.3%)	9 (90.0%)	12 (92.3%)
Lidocaine + adrenaline, *n* (%)	18 (78.3%)	8 (80.0%)	10 (76.9%)
Sufentanil adjuvant, *n* (%)	6 (26.1%)	2 (20.0%)	4 (30.8%)
Dexamethasone adjuvant, *n* (%)	7 (30.4%)	3 (30.0%)	4 (30.8%)
NSAID adjuvant, *n* (%)	7 (30.4%)	3 (30.0%)	4 (30.8%)
Supplemental O_2_ required, *n* (%)	2 (8.7%)	1 (10.0%)	1 (7.7%)

IQR = interquartile range; ASA = American Society of Anesthesiologists Physical Status Classification; NSAID = non-steroidal anti-inflammatory drug.

**Table 2:** Summary of haemodynamic monitoring parameters, dosing variables, and satisfaction outcomes with Spearman rank-order correlation coefficients relative to total dexmedetomidine dose.

Parameter	Median (IQR)	Range	Spearman ρ vs. Dose (*p*)
Total dexmedetomidine dose, μg	52 (44–68)	15–108	— (reference)
Sedation-to-incision interval, min	50 (40–75)	10–96	ρ = 0.74 (< 0.001 *)
Duration of surgery, min	45 (30–55)	10–80	ρ = 0.29 (0.19)
Body weight, kg	66 (56–85)	46–113	ρ = 0.43 (0.039 *)
Age, years	76 (67–80)	50–85	ρ =− 0.17 (0.44)
BMI, kg/m^2^	24.6 (22.5–30.0)	16.6–38.2	ρ = 0.37 (0.083)
Baseline systolic BP, mmHg	152 (130–178)	91–190	—
Nadir systolic BP, mmHg	98 (90–120)	72–135	—
Decrease in systolic BP, mmHg	38 (25–55)	8–100	ρ = 0.002 (0.99)
Baseline heart rate, beats/min	65 (57–77)	52–93	—
Nadir heart rate, beats/min	52 (45–60)	43–79	—
Decrease in heart rate, beats/min	12 (8–18)	3–29	ρ = 0.31 (0.15)
Patient NRS satisfaction (1–10)	10 (10–10)	4–10	ρ =− 0.27 (0.22)
Surgeon NRS satisfaction (1–10)	10 (10–10)	6–10	ρ = 0.08 (0.73)

IQR = interquartile range; BP = blood pressure; NRS = Numerical Rating Scale (1–10). * Statistically significant at α = 0.05 (two-tailed).

## Data Availability

The raw data supporting the conclusions of this article will be made available by the authors upon reasonable request.
